# Sanitation

**Published:** 1889-08

**Authors:** J. P. Stevens

**Affiliations:** Macon, Ga.


### Southern Medical Record.

A MONTHLY JOURNAL OF PRACTICAL MEDICINE.

All communications must be addressed to R. C. WORD, Managing Editor.

"*Quicquid Præcipies Esto Brevis.*"

VOL. XIX ATLANTA, GA., AUGUST, 1889. NO. 8

### ORIGINAL AND SELECTED ARTICLES.

### SANITATION.

By J. P. Stevens, M. D., Macon, Ga.

Read before the Macon Athenæum.

A higienist has recently delivered a lecture before a Board of Education in Ohio, and though a layman, he occupies a distinguished position as a superintendent of instruction of a large number of children and youth; and as Bacon, many years ago, declared a sound mind in a sound body to be the greatest blessing that could be conferred upon a man, the superintendent selected as the subject of his lecture, "The Contagion of Health." This, at first sight, appears to be a novel subject, for the majority of mankind are so regardless of the laws of health, that, even if they are acquainted with them, such laws are often treated with slight consideration if not with contempt. The superintendent, however, with, I might say, appalling significance, delineates the intellectual armature of the modern medical practitioner. He says: "I have been credibly informed that the modern medical practitioner must be prepared with the latest methods of staining the tubercular baccillus; must be able to diagnose under the microscope an alveolar carcinoma from an alveolar cancer, should be accustomed to recognize with the opthalmoscope the earliest stage of optic atrophy, and to diagnose with the laryngoscope paralysis of one vocal cord, to describe fully the electrical reactions of a degenerated muscle, and withal to keep accurately in mind the kariokinesis of a nucleus, and the life history of filaria sanguinis hominis, and the dissolution of a fibroid by electricity; and this is not telling the half of it." You ought not, therefore, to be astonished at the self-assumed consequentialness of certain modern disciples of Æsculapius, when the public accredits them with the possession of such transcendent wisdom. Bill Nye, however, seems to differ somewhat from the above inflated eulogy of the medical man in his criticism of his own doctor. He says: "He was a good doctor—for horses; but he was out of his sphere when he strove to fool with the human frame. Change of scene and rest were his favorite prescriptions. Most of his patients got both, especially rest; a long, unbroken rest. That is his specialty. He did not know what was the matter with me, but he seemed willing to learn." But in all seriousness, you must bear in mind that the doctor is at all times confronted with the great problem of life. The innermost aspiration of his soul is to save life. His meat and his drink, even his nocturnal dreams are associated with the anxious inquiry, how shall I prolong the greatest boon to humanity—life?

Now what is life? According to Herbert Spencer, "life is a definite combination of heterogeneous changes simultaneous and successive in correspondence with external coexistences and sequences." This you perceive is a very lucid definition, clearly comprehensible by the most obtuse intellect. But even assuming, as a matter of course, that in this elite association there are no obtuse intellects, and cognizant of the fact that the remote so-called fixed stars are without the range of a telescope of 1,000 diameters, I will presume to paraphrase the above definition thus: Life is an adjustment or correspondence of our external relations to our internal relations; a correspondence of the functional activities of our bodily organs, with their environments or surroundings. Now, every day we are brought in relation to physical, ethical, esthetic, social and religious influences and conditions that we cannot escape, and which are under the control of the general laws of evolution. These we call our environments, which we cannot avoid, and with which we must bring our internal relations in correspondence. So varied and complex is the machinery that controls the vital functions, its processes are styled a microcosm, a little world wherein we find analagous phenomena to those concerned in the various mechanical industries. First, there is an organ the special office of which, like the bellows of a smith's forge, which, at 20 respirations per minute, furnishes the fuel necessary to produce the heat-force required to drive the machinery, then there is a powerful force-pump with energy equivalent to four and one half pounds for impulse that, seventy-five times per minute, drives the vital fluid through its innumerable conduits into the remotest parts of the body; then there is the great receptacle of the raw material which is ground up into a milky fluid and put into a suitable condition for transference through the distributing canals finally to be worked up into textile fabrics of the most wonderful diversity and complexity. For this purpose a set of mechanics are detailed whose duty it is to separate from the conglomerate mass that which is constructive and conservative, and another set whose special function is to eliminate from the crude material that which is degenerated and destructive, and convey them out of the body. So you perceive we have an assimilation to the operations of two companies of workmen engaged upon a building; on the one side those engaged in building up, those on the other side employed in pulling down. It is readily comprehended that there must be an equilibrum maintained in these two processes. Excess on either side must be followed by loss. If the degenerative process exceed the recuperative, whether in health or disease, ultimate death will sooner or later be the inevitable consequence unless the equilibrium is re-established. Now, at the head of all this complex machinery, and directing the movements of the associated organs, we find the brain with its vast system of voluntary and sympathetic nerves, like an intricate net-work of telegraph wires, directing and co-ordinating the movements of the vital functions. Associated with the wants of the five senses by which we are brought in direct relation to the external world, there are ethical, social, mental, religious and emotional activities, all tending to the consummation of happiness or misery. Harmonious and reciprocal activity of the vital functions and the faithfulness with which we bring our external relations in adjustment with our internal relations are the conditions of health. Now what are these external relations? Pure air, which is supplied by nature in the greatest abundance and with molecular constituents in kind and quantity to suit every physical want. Atmospheric air under adverse conditions is the most prolific source of disease, as it is the carrier of contagion induced by uncleanliness, individual, domestic and municipal. The human body is composed in part of 80% of water, hence this fluid comes next in the category of physical wants, along with plenty of wholesome digestible food. To successfully utilize these beneficent gifts of nature, personal and municipal cleanliness and the most thorough domiciliary ventilation must be obtained, that the air may not be contaminated by microbes through the fermentation of animal and vegetable debris; pure water, also devoid of the same contamination; plenty of outdoor exercise which will usually procure refreshing sleep so necessary to the recuperation of exhausted energy, and which is best secured by observing the old trite maxim, "early to bed and early to rise, &c."

These are the external relations with which if we live in close correspondence, will secure to us the highest degree of attainable happiness. Let us ever bear in mind that the presiding genius who in queenly majesty controls and co-ordinates these diverse relationships, and secures to us the ineffable boon is temperance. Temperance in drinking, eating, sleeping, working, dancing, studying, loving and talking as well.

How few there are who observe the laws of health with the sagacity which we so often see among inferior animals. They rigidly follow the dictates of instinct in the gratification of their appetites, and rarely exceed the bounds of moderation unless under adverse and unusual conditions. Hence they uniformly, under favorable environments attain to the age which relatively would warrant to man the expectation of a climax of 100 years, with the observance of similar habits of caution and circumspection. The mutual relationships that subsist between the mental and physical faculties, and the interdependence of their functional activities, demand an intelligent acquaintance with, and a scrupulous observance of the laws which control the forces of nature in their wonderful correlations and interactions. There is no inhabited domicile, town or city, which will not so overburden the natural processes of purification as to make some provision necessary for counteracting the degenerative waste of living. There is a never ceasing conflict for the mastery between life and death, from the first breath of the infant to the last groan of the centenarian; from the first rupture of the cell-wall of the plant germ to the final dismantling of the forest giant of 1,000 years. There are conflicts within us, conflicts without, conflicts all around. There are often germs of death in the cooing of an infant, the merry laugh of the romping school girl, or the melodious trill of the professional cantatrice; in the gentle undulations of the purling brook or the mighty swell of the majestic river that bears upon its bosom the commerce of nations.

Independently of our personal responsibilities as concerns our individual relationships to external conditions, we can not be blind to the responsibilities of municipal corporations, as well as to the duties of the State and Nation for the conservation of the public health. Pure water, pure air and pure food are the foundation upon which rest the fair temple of Hygea. In stately royalty she wields an imperial sway, and unequivocally demands a compliance with her behests. The decomposition of animal and vegetable debris, heat and moisture, are the rueful sources of all the maladies which afflict humanity. The scrupulous removal of all animal and vegetable offal, an inexorable warfare against dirt, personal and external, through soil and subsoil, drainage, an efficient system of sewerage, thorough ventilation of every nook and corner of our living rooms as well as our sleeping apartments, the cooking of food in accordance with correct chemical and physiological principles, and well regulated out door bodily exercise, are attainable conditions of health. The careless and imperfect preparation of food is one of the most prolific sources of bodily maladies which consigns to premature graves vast multitudes of human beings from every class of society. How necessary therefore, that the principles of sanitation should be taught around our firesides and illustrated at our meals, as well as be the subject of scholastic and public instruction ; for without health all earthly good is " vanity and vexation of spirit." For the attainment and preservation of health one of the most valuable co-operatives is a joyous and contented social disposition. A despondent temperament that ever darkens the future with shadowy portents, and is ungrateful for present blessings, is perpetually wearing away the machinery of existence and consigning it to premature dissolution. It has been truly declared : " If he is a public benefactor who makes two blades of grass to grow where one grew before, what is he who makes a flower of joy spring from the grave of a groan.

Ella Wheeler Wilson in writing to the great humorist Marshall P. Wilder, thus addresses him :

" If some day you should meet with Trouble,
However frowning fierce and grim,
I'm sure you'd bend the monster double
With laughter, e'er done with him
And care would hide her face and smile,
If once she talked with you awhile.
So much life holds to wake our tears,
God bless the man who rouses laughter.
For him, whatever warms and cheers,
On Earth, and Heaven's best crown hereafter.
So jest away—Dear little friend—
Keep the world smiling to the end."

Let us therefore heed the lessons of wisdom and practice her precepts. Then why may we not attain the green old age of the great French chemist Chevreul, who, a few months ago, celebrated his 102 anniversary? On that occasion he was complimented by a lady on his remarkably well preserved appearance. He replied: "Madam, you are very kind, but I am going down the hill. What would I give if I were only 80."

